# The Care of the Baby

**Published:** 1896-03

**Authors:** 


**The Care of the Baby.** By J. P. Crozer Griffith, M.D. Pp. 1-8, 17-392. Philadelphia: W. B. Saunders. 1895.

In writing a hand-book for mothers and nurses without confusing his readers with scientific words, Dr. Crozer Griffith has succeeded where many others have failed. From cover to cover his book contains excellent advice tempered with sound common sense, and we sincerely hope that it will have the wide circulation it deserves; for the mother in the United States seems to be as ignorant as the one on this side of the water.

The book is well printed; but it was hardly necessary to give a picture of a knitted sock or of a bassinet.

